# The effect of collectivism-oriented human resource management on employee resilience of hospitality employees

**DOI:** 10.3389/fpsyg.2024.1342318

**Published:** 2024-05-03

**Authors:** Shihua Chen, Xiaohan Hu, Yuting Xue, Yuang Wang

**Affiliations:** ^1^SILC Business School, Shanghai University, Shanghai, China; ^2^College of Shanghai Lausanne Hospitality Management, Shanghai Business School, Shanghai, China

**Keywords:** collectivism-oriented human resource management, the group engagement model, perceived overall fairness, social identity, employee resilience

## Abstract

**Introduction:**

In the face of an increasingly challenging and rapidly evolving business environment, not all the employees exhibit the requisite resilience necessary to recover from adversity. From both the individual and organizational perspectives, enhancing employee resilience emerges as a critical issue not only in the practical and academic fields. In the Chinese culture, this research aims to investigate how and why collectivism-oriented human resource management (C-HRM) fosters employee resilience. Drawing on the group engagement model, we propose a serial mediating effect of perceived overall fairness and three dimensions of social identity between C-HRM and employee resilience.

**Methods:**

Using a sample of frontline employees in the hospitality industry, we conducted a field survey among 342 employees (study 1) and a two-wave online survey among 294 hospitality employees (study 2).

**Results:**

Findings from empirical analysis indicated that C-HRM significantly increases overall fairness perception of hospitality frontline employees and in turn, their identification and respect, which further fertilize employee resilience. In addition, the indirect effect of C-HRM on employee resilience through perceived overall fairness and pride was not statistically significant.

**Discussion:**

These important findings are expected to help employees cope with the workplace pressures caused by ongoing challenges and change, and contribute to sustainable career development.

## 1 Introduction

In today’s era, characterized by constant challenges and rapid changes, the intricate and volatile nature of organizational environments significantly influences how employees perceive, structure, and execute their work (Minbaeva and Navrbjerg, 2023). For instance, the increasing prevalence of the organizational downsizing, restructuring, and job redesign has introduced uncertainty regarding employees’ financial stability, career prospects, and daily work routines. Consequently, this has engendered substantial pressure among employees, diminishing their motivation and culminating in emotional exhaustion and job burnout (Lu et al., 2023). Employee resilience plays a pivotal role in addressing unforeseen changes and crises, as individuals possessing strong employee resilience exhibit various positive attributes, such as optimism, confidence, energy, enabling swift self-regulation to better contend with job-related stressors (Malik and Garg, 2017; Thompson et al., 2018). Studies have shown that resilience can significantly enhance employee satisfaction (Youssef and Luthans, 2007), organizational commitment (Meng et al., 2019), and employee engagement (Cooke et al., 2019). Therefore, nurturing and maintaining resilience to confront challenges and adapt to the evolving work environment is critical for safeguarding employee wellbeing and ensuring organizational sustainability. For frontline employees in the hospitality industry, it is particularly significant to enhance employee resilience. These employees are known for enduring low wages, long working hours, job instability, and poor employment conditions (Pienaar and Willemse, 2008; Zhang et al., 2017). Additionally, emotional labor occupies an integral part of job demands of hospitality frontline employees, exacerbating their psychological burnout and emotional exhaustion (Shapoval, 2019). Therefore, we aim to explore how to create, sustain and promote employee resilience for hospitality frontline employees, thereby maintaining their wellbeing and job performance in times of change and challenge.

Given that resilience is a malleable capacity, it can be cultivated through interventions and may be responsive to effective management practices (Britt et al., 2016). Several organizational management practices have been identified by researchers as playing a crucial and positive role in fostering work resilience, including sustainable human resource management (Lu et al., 2023), happiness-oriented human resource management (Cooper et al., 2019), high performance work systems (Cooke et al., 2019), and high engagement work practices (Meacham et al., 2023).

Despite the findings of employee resilience in the hospitality management literature, how culture factors affect employee resilience is limited. It is noteworthy that organizations in different geographical or cultural contexts tend to exhibit distinguishing human resource management (HRM) characteristics (Hofstede, 1993; Aycan et al., 2000; Gerhart and Fang, 2005; Farndale, 2010; Katou et al., 2010; Rode et al., 2022). Hence, researchers call for more research on the influence of culture related leadership and human resource practices on employee resilience (Wright et al., 2008; Donaldson et al., 2020; Ashraf et al., 2022; Tong et al., 2022; Zhang et al., 2024). One of the typical cultural factors commonly studied in China is collectivistic culture.

Collectivism, a key cultural characteristic deeply rooted in Confucianism, represents a significant difference between oriental culture and other cultures (Oyserman et al., 2002). The integration of collectivistic cultural values and strategic HRM gives rise to collectivism-oriented human resource management (C-HRM), which is defined as a set of human resource management policies or practices that shape a collectivistic culture within an organization (Li et al., 2012). Previous research has shown that C-HRM can enhance employees’ creativity and performance, thereby contributing to the achievement of the organization’s strategic objectives (Li et al., 2012; Chen et al., 2016, 2021). However, to date, the research on C-HRM is still in its infancy, and predominantly focuses on stimulating employees’ proactive behavior and improving organizational performance. To the best of our knowledge, studies on C-HRM and employee resilience are scarce in the research literature. Therefore, the relationship between C-HRM and employee resilience requires further study. In order to address this research gap, we explore the impact of C-HRM on employee resilience in the context of Chinese management.

**FIGURE 1:**
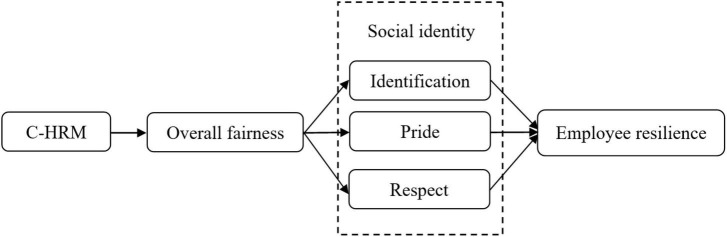
The conceptual model.

Therefore, the objectives of this study are (1) test the relationship between C-HRM and employee resilience; (2) test the mechanism between C-HRM and employee resilience. Specifically, we propose a research model (See Figure 1) drawing on the group engagement model. The group engagement model elucidates why people attach significant importance to justice and how they integrate their self-concept with their group to form social identity, subsequently acting on behalf of the group (Tyler and Blader, 2003). Additionally, the research shows that the group engagement model, which emphasizes group concerns, is more applicable to collectivistic values than individualistic values (Ma et al., 2016). Therefore, we take the group engagement model as a theoretical framework to link C-HRM to employee resilience and introduces organizational fairness and social identity as chain mediating variables. Specifically, C-HRM facilitates employees in focusing on the consistency and similarity among organization members rather than individual uniqueness, thus reducing perceived differences among individuals and enhancing the perception of fairness. Based on the group engagement model (Tyler and Blader, 2003), organizational justice conveys positive information about employees’ identity and status, thereby reinforcing their positive social identity within the organization. Motivated to maintain and promote a positive self-concept, employees with a positive social identity in the organization take the initiative to develop employee resilience and respond well to pressure, challenges, and crises to achieve personal and organizational goals.

Based on the factors above, the present study holds significant importance for academic research and management practice in several aspects. First, this study is useful in enriching the research on employee resilience. In the past, researchers and managers have overemphasized job performance and largely neglected positive mental state of employees, such as employee resilience. Recently, studies have begun to pay attention to the resilience of healthcare workers (Labrague, 2021), and identified several antecedents of resilience, including leadership style, social support, work feedback, and organizational management practices (McDonald et al., 2016). However, empirical studies on frontline employees’ resilience in the hospitality industry remain insufficient. It is noteworthy that frontline service employees, especially in the face of complex and evolving environments, require a high level of employee resilience to swiftly recover from the challenges and pressures inherent in their roles. Therefore, it is of great theoretical value to investigate how HRM practices may improve hospitality frontline employees’ resilience in the Chinese context.

Second, this study is conducive to drawing the attention of academic researchers and business practitioners to C-HRM, thereby enriching C-HRM research. Chinese society is facing a fiery management reform, Chinese-style management has risen to prominence aroused strong attention. However, C-HRM, a management issue with distinct oriental characteristics, remains relatively underexplored in the decade since it was introduced. Existing research on C-HRM is very limited, impeding the advancement of research in this domain. Given that collectivism is a prominent cultural value in China and many East Asian countries, coupled with the globalization of business activities and the frequent occurrence of mergers and acquisitions, this study not only contributes to C-HRM but also holds significance for international HRM research.

In addition, the findings of this study provide implications of relevance to management practitioners. This study identifies some important new roles for C-HRM. Since human resource is a valuable resource for business success and can be a sustainable source of competitive advantage, fostering employee resilience not only improves sustainability of career development, but is also critical to business survival and growth. As this study demonstrates, the adoption of C-HRM may improve employee resilience by enhancing perceived overall fairness and social identity. Therefore, C-HRM can be viewed as a strategic initiative for firms and may exert great value in volatile, complex, and competitive environments.

## 2 Literature review and hypotheses

### 2.1 C-HRM and perceived overall fairness

Fairness in the workplace is an important issue in HRM and various research have confirmed that individual thoughts, feelings, and behaviors are shaped by organizational justice (Hang-yue et al., 2006; Patel et al., 2012; Abuelhassan and AlGassim, 2022; Li et al., 2022). In previous studies, most scholars have believed that organizational justice is reflected in three dimensions, namely distributive justice, procedural justice and interactional justice, with focus on the differentiation of specific dimensions of justice (Colquitt, 2001). Notably, the emphasis on specific dimensions of organizational justice has limitations, since the specific dimensions of justice do not function in isolation (Ambrose and Schminke, 2009). When individuals form the perception of fairness, they are making a holistic judgment which ultimately drives their behaviors (Greenberg, 2001; Patel et al., 2012). As a result, individual experience of fairness in the workplace may not be fully and accurately represented by specific forms of fairness, and more research should be done on overall fairness to provide a comprehensive picture of how individuals assess and use fairness judgment. Perceived overall fairness refers to employees’ global assessment of organizational fairness based on individual experience and the experience of others (Ambrose and Schminke, 2009). In view of the importance of organizational fairness, numerous antecedents of employees’ perceived fairness in the workplace have been identified, including personality traits, leadership, team characteristics (Colquitt et al., 2002) and organizational management practices (Cohen-Charash and Spector, 2001; Balser, 2002). Besides, cultural values are also identified as important determinants of organizational justice (Conner, 2003; Primeaux et al., 2003; Hang-yue et al., 2006).

Research on collectivism is a vital topic in cross-cultural literature. Organizational management practices present unique characteristics based on different socio-cultural contexts, thereby the existence of collectivistic culture affects organizational climate and HRM practices in a variety of ways (Ramamoorthy et al., 2007). C-HRM is a series of HRM policies and practices that cultivate the collectivistic cultural values in the firm (Li et al., 2012), aiming to imperceptibly guide employees’ attitudes and behaviors to achieve organizational goals by shaping collectivistic values within the organization (Chen et al., 2016). Several studies conducted with East Asian organizations as samples have reached meaningful conclusions. For example, C-HRM has been proved to be an effective HRM system with numerous positive effects on employees and organizations, including substantial improvements in job satisfaction, reduction in turnover intention (Li et al., 2014), enhancement of employees’ innovation (Chen et al., 2016), contribution to team creativity (Chen et al., 2021), and improvement in organizational performance (Li et al., 2012). However, the number of such studies is still relatively limited, and the outcome variables investigated in these studies are relatively simplistic. Therefore, it is necessary to conduct in-depth research on C-HRM both in empirical data and theoretical pathways.

In organizations implementing C-HRM, collectivistic cultural values penetrate into every activity of HRM, significantly influencing individuals’ perceptions, attitudes, behaviors, and social relationships (Chen et al., 2015, 2016, 2021). For instance, C-HRM focuses on the consistency of the whole team rather than the particularity of individuals in performance evaluation (Papalexandris and Panayotopoulou, 2004). The employee selection and promotion tend to be on the basis of the cooperative ability and loyalty of employees rather than on the basis of personal attributes (Triandis, 1995). In employee training, emphasis is placed on teamwork and the shaping of collective consciousness rather than the improvement of individual competence (Li et al., 2014). These group-focused HRM practices enables employees to maintain interpersonal harmony by being consistent with other members of the organization, strengthen common values, and achieve common goals (Goncalo and Staw, 2006).

Consequently, C-HRM underlines a focus on interpersonal similarity and team consistency, which guides employees’ perceived overall fairness (Gedik and Ozbek, 2020). Specifically, influenced by C-HRM, employees perceive and think more in terms of “we” and pay attention to the similarities and congruence between self and coworkers, resulting in individual differences in rewards, opportunities and interpersonal treatment are overlooked. Thus, employees under C-HRM experience less prejudice, discrimination and inequality that increases overall fairness perception. In line with this view, Nyaw and Ng (1994) and Hang-yue et al. (2006) showed that collectivists perceive less prejudice and discrimination in the workplace, as they make fewer social comparisons and are more tolerant and less sensitive to inequalities among colleagues. Erdogan and Liden’s (2006) study similarly found that collectivists have greater tolerance for unfairness. To sum up, we argue that C-HRM emphasizes the similarity and consistency among organizational members, which weakens individual perception of differences and in turn improves perceived overall fairness. Therefore, we propose the following hypothesis:

Hypothesis 1: C-HRM is positively related to perceived overall fairness.

### 2.2 The mediating role of perceived overall fairness

Social identity is a part of individual self-concept deriving from people’s perception of themselves as members of a group and the value and emotional significance attached to such membership (Tajfel, 2010). The group engagement model (Tyler and Blader, 2003) views social identity as a multidimensional construct that contains a cognitive component and two evaluative components. Identification refers to the extent to which individuals combine self-awareness with groupthink, see themselves and their group in similar ways, and define themselves in terms of their group membership. In other words, identification captures employees’ cognitive belief that they belong to and are integrated with the organization (Ashforth and Mael, 1989; Blader and Tyler, 2009), and emphasizes belongingness and oneness with the organization. Pride and respect constitute the evaluative components of social identity and serve as status indicators. Pride reflects the employee’s evaluation of the organizational status, and respect is the employee’s evaluation of his or her status in the organization (Tyler and Blader, 2003; Blader and Tyler, 2009). In social identity research, identification is typically the primary focus (Ashforth and Mael, 1989; Olkkonen and Lipponen, 2006), while evaluative components have received little attention. The neglect of evaluative components in social identity research indicates an incomplete understanding of the structure of social identity. Because the evaluative components of social identity reflect individual judgments about the value of group membership, determining the importance of group membership in how individuals think about and perceive themselves, which is essential to understanding the concept and impact of social identity (Blader and Tyler, 2009; Tajfel, 2010). Therefore, it is necessary to clarify the definition of social identity in research and conduct further studies on the evaluative components of social identity, namely pride and respect.

HRM practices play a crucial role in shaping the social identity of organizational members, specifically by conveying the establishment and interpretation of organizational norms and values to employees through HRM activities such as communication and training (Besharov, 2014). C-HRM is rooted in collectivistic cultural values which is closely related to social identity (Brickson, 2007). In collectivistic cultural values, the organization is usually regarded as a “big family.” Employees are not only in an exchange relationship with the organization, but also in a family-like and mutually supportive relationship, which makes it easier for employees to develop positive perceptions and emotional attachments to the organization (Kim and Coleman, 2015). Therefore, C-HRM affects employee social identity mechanism toward the organization.

First, C-HRM is beneficial to foster identification. C-HRM advocates building a harmonious relationship between the organization and employees, aiming to cultivate employee’s self-concept of as part of the organization (Brickson, 2007; Li et al., 2014). Under the influence of C-HRM, employees are inclined to define themselves as members of the organization and develop a strong psychological attachment to the organization (Triandis, 1995; Hong et al., 2016). This connection between employees and the organization facilitates employees’ identification with the organization as they perceive themselves as closely related to the organization and associate their self-concept with the organization. Liu et al. (2013) provides empirical data to support this view that C-HRM promotes organizational identification. Second, C-HRM fosters employee pride. C-HRM encourages efforts to comply with organizational norms, internalize organizational values, and show strong organizational loyalty, thus leading employees to evaluate their organization from a positive perspective (Triandis, 1995; Hong et al., 2016). Employees’ belief that the organization has attractive values can lead them believe that they work in a prestigious organization with high social status, which in turn inspires a sense of pride (Tyler and Blader, 2003; Fuller et al., 2006). In addition, C-HRM can also foster employees’ feelings of being respected in the organization. C-HRM emphasizes teamwork and interpersonal harmony (Li et al., 2012). By improving team cooperation and enhancing communication and coordination, C-HRM helps employees gain more understanding and support from colleagues in their work interactions (Chen et al., 2016). Therefore, C-HRM allows employees to feel accepted and valued by other organizational members rather than rejected and excluded, providing them with a sense of respect. Based on the above, we argue that C-HRM has a positive impact on all three dimensions of social identity, i.e., identity, pride and respect.

Drawing on the group engagement model, fairness is an organizational prerequisite for social identity (Tyler and Blader, 2003). Fairness shapes judgments about individual identity and status as a group member. Specifically, individuals tend to utilize perception of fairness to evaluate the quality of interpersonal treatment they receive in a group, which conveys key messages about status (Tyler and Blader, 2003). When people feel fairly treated by the group, they perceive their group as having high status as well as themselves as having high status within the group, and integrate their self-concept with the organization to create a positive self-identity. Employees create a positive evaluation of their social self and gain a sense of worth and self-esteem through group membership (Tyler and Blader, 2000). Thus, perceived overall fairness positively influences employee social identity in the organization.

Next, we will elaborate the impacts of perceived overall fairness on identification, pride, and respect, respectively. Perceived overall fairness shapes employee perception of organizational membership. Drawing on the group engagement model (Blader and Tyler, 2009), fairness provides organizational members with identity security, indicating that they can safely derive self-consciousness from the organization. It attracts employees to form a cognitive connection with their organization, and define themselves in terms of organizational membership. Employees who are cognitively aware that they are part of the organization and believe that organizational membership is emotionally significant will develop identification with the organization (Tajfel, 2010). In addition, research has shown that organizational justice affects employees’ belief about their legitimacy as organizational members (Dutton et al., 1994; Ashforth et al., 2008). When employees believe they are legitimate members of the organization, they tend to be psychologically and emotionally attached to the organization (Shen and Benson, 2016). Therefore, perceived overall fairness promotes identification by enhancing the extent to which employees cognitively integrate their self-concept with the organization.

Perceived overall fairness conveys status-related messages, including pride and respect. Fairness is the embodiment of organizational prestige which signifies organizational social status. Drawing on the group engagement model, fair treatment by the organization causes employees to evaluate their organization in a positive light and thus perceive the organization to which they belong as having a high status (Tyler and Blader, 2003; Fuller et al., 2006). The social status of the organization is a potential source of self-construal for organizational members (Dutton et al., 1994), playing an important role in influencing employee pride (Carmeli and Tishler, 2004; Helm, 2013). If the organization enjoys a good reputation and prestige, it will carry over to individual self-esteem and self-worth and bring about a sense of pride (Tajfel, 2010). Previous studies have shown that procedural justice, distributive justice, interpersonal justice and informational justice have a positive impact on pride (Pereira et al., 2021). Therefore, perceived overall fairness elicits a high sense of pride from the organizational membership.

Additionally, organizational fairness symbolizes individual status in the organization. Drawing on the group engagement model (Tyler and Blader, 2003), employees tend to use perceived fairness to assess their status in the group in order to increase their sense of self-worth. Higher status in the organization means more respect, while lower status in the organization means less respect. Specifically, when employees are treated with courtesy, equality and dignity by the organization, they believe that they are accepted, recognized and valued as part of the organization, thus inferring that their status in the organization is affirmed and experiencing a high level of respect (Tyler and Lind, 1992). In contrast, when the organization treats employees in an unfair manner, they are possible to speculate that their status in the organization is undermined and questioned, thus feeling a lack of respect. Consistent with this argument, prior research has shown that procedural justice, distributive justice, and interactional justice are prerequisites for respect (Spence Laschinger, 2004; Hart et al., 2019). Therefore, perceived overall fairness fertilizes respect by leading employees to believe that they have a high status in the organization.

Given that we postulate the impact of C-HRM on perceived overall fairness (Hypothesis 1), and separately demonstrates the impact of C-HRM and perceived overall fairness on the three dimensions of social identity (i.e., identification, pride, and respect), we argue that perceived overall fairness plays a mediating role in the relationship between C-HRM and social identity. Specifically, C-HRM improves perceived overall fairness by emphasizing similarity and consistency among team members. Employees further utilize perceived overall fairness as a clue to infer their organizational membership and status. When employees feel fairly treated by the organization, they feel a state of respect and pride, and integrate their self-concept with the organization. Therefore, we propose the following hypotheses:

Hypothesis 2a: Perceived overall fairness mediates the relationship between C-HRM and identification.

Hypothesis 2b: Perceived overall fairness mediates the relationship between C-HRM and pride.

Hypothesis 2c: Perceived overall fairness mediates the relationship between C-HRM and respect.

### 2.3 Social identity and employee resilience

Resilience is a positive psychological ability that empowers individuals to “bounce back” and even positively change, progress, and surpass from adversity, uncertainty, conflict, failure, and increased responsibility (Luthans, 2002). In the context of the workplace, employee resilience is an ability to effectively manage daily job challenges, including navigating work-related stress to maintain wellbeing, learning from unexpected setbacks, and actively preparing for future challenges (McEwen and Boyd, 2018). Employees with a high level of employee resilience can proactively and swiftly mobilize their positive psychological resources to reinvigorate from stressful events, learn and grow from difficulties, and not only return to their previous level but also emerge stronger than before in the face of significant changes, adversity, or risks at work (Meneghel et al., 2016). Existing research has examined many factors that positively influence employee resilience, such as leadership style (Peng et al., 2022; Cai et al., 2023; Mao et al., 2023), social support (McDonald et al., 2016; Kuntz et al., 2017), and organizational climate (Meneghel et al., 2016; Malik and Garg, 2017). These studies can be categorized into two research streams. Drawing on the conservation of resources theory, positive work environments, work events, and leadership styles can function as positive work resources, which are important sources of individual resilience (e.g., Meneghel et al., 2016; Kuntz et al., 2017; Cai et al., 2023). Another research stream posits that, drawing on the principle of reciprocity and social exchange theory, employees are more likely to engage in resilient behaviors when organizations or leaders ensure employees’ perceived support, exchange quality, emotional commitment, trust, and psychological contract fulfillment (e.g., Meng et al., 2019; Zhu et al., 2019; Mao et al., 2023). However, there is limited research on the relationship between social identity and employee resilience. Previous literature has suggested that social identity can serve as a key factor in protective buffering during a crisis (Drury et al., 2008; Templeton et al., 2020). Therefore, the potential impact of social identity on employee resilience deserves further exploration.

Social identity is a key determinant of important psychological and behavioral connections between individuals and organizations. According to the group engagement model (Blader and Tyler, 2009), willingness and behavioral efforts of organizational members to exhibit group engagement are influenced by the role that the organization plays in shaping their self-concept. Individuals create a positive social self by cues about identity and status from the organization. Maintaining and improving such positive social self is a core motivation for them to participate in the organizational development process (Tyler and Blader, 2003; Tyler, 2012). People will contribute more discretionary effort to the organization with positive identity cues because association with this organization builds a positive identity and ensures that they identity with an organization that makes them feel well about their social self in order to maintain a positive self-concept (Tyler and Blader, 2003). It can be concluded that social identity may contribute to the improvement of employee resilience. To be specific, when people integrate their identity with the organization, perceive the organization as high status, and receive respect within organization, they are more likely to be energized by the motivation to maintain and improve their positive self-concept, showing greater resilience at work to better fulfill job demands and advance organizational goals.

Identification has a positive impact on employee resilience. Drawing on the group engagement model (Tyler and Blader, 2003), identification creates a solid bond of connection between the employee and the organization, determining the extent to which s/he form supportive attitudes and behavioral engagement in the group. Individuals with strong organizational identification integrate their self-concept with the organization, and view the organization’s success as their own (Ashforth and Mael, 1989). For them, an intrinsic concern is developed that motivates them to have a higher sense of responsibility and loyalty to the organization, and engage in group activities to support the organizational interests and achieve organizational goals. Thus, identification provides intrinsic motivation for employees to proactively improve their employee resilience to cope with stress and frustration and to act more positively in challenging situations. Supportive evidence for this opinion is offered by existing research that suggests that organizational identification is a positive predictor of employee resilience (Lyu et al., 2020; Srivastava and Madan, 2020; Peng et al., 2022; Mao et al., 2023). Therefore, we propose the following hypothesis:

Hypothesis 3a: Identification is positively related to employee resilience.

Pride has a positive impact on employee resilience. The group engagement model argues that status judgments associated with the organization can shape group engagement as the organization play an important role in creating and maintaining positive personal identities, (Tyler and Blader, 2003). Specifically, individuals derive their self-image from the organization to which they belong, and organization membership can enhance or degrade individual self-concept (Tajfel, 2010). Pride implies that employees believe their organization has a high social status, which answers the question “how am I perceived by people outside the organization” (Dutton et al., 1994). Employees who are proud of their work and organization are aware of the positive identity hints provided by organizational membership, such as a sense of worth and reputation, thus valuing the organization’s external prestige and striving to improve the organizational status to maintain a positive social self (Tyler, 1999). Hence, employees can be empowered by pride to improve employee resilience, rise to the occasion and exceed expectations in order to promote organizational success. Therefore, we propose the following hypothesis:

Hypothesis 3b: Pride is positively related to employee resilience.

Respect has a positive impact on employee resilience. Respect reflects individual belief that s/he is a valuable member of the organization and enhances group member’s efforts to contribute to the achievement of group welfare and goals (Tyler and Blader, 2000). The group engagement model suggests that respect draws employees’ attention to their unique value, leading them to dedicate their unique thinking and engagement to valuable creative actions to achieve organizational goals aimed at solidifying their favorable position as organizational members by reinforcing their positive image in the organization (Tyler and Blader, 2003). Therefore, in order to maintain a positive self-concept and enhance self-worth, employees who feel respected by others in the organization will remain enthusiastic about the work, take the initiative to exert their specific capacities (Parker et al., 2010; Ekrot et al., 2016), and search for solutions to overcome stress and adapt to uncertainty by a variety of methods, thus showing greater employee resilience to better handle of unexpected threats and crises (Chang et al., 2012). On the contrary, employees who lack respect perceive themselves to be at a disadvantage in the organization and are skeptical of their importance and value, thereby displaying negative work attitudes and ineffective work behaviors (Tyler, 1999; Tyler and Blader, 2000). Such employees are less resilient at work and have a greater likelihood of engaging in withdrawal behaviors in the face of adversity rather than adopting a positive attitude to respond proactively. Existing studies have also shown that respect is significantly related to self-improvement motivation, promoting individual self-development (e.g., Fuller et al., 2006). Therefore, we propose the following hypothesis:

Hypothesis 3c: Respect is positively related to employee resilience.

### 2.4 The serial moderating role of perceived overall fairness and social identity

So far, we have hypothesized indirect effects of C-HRM on the three dimensions of social identity (i.e., identification, pride, and respect) by affecting perceived overall fairness, as well as direct relationships between the three dimensions of social identity (i.e., identification, pride, and respect) and employee resilience. Synthesizing the above reasoning, we further hypothesize that the indirect effect of C-HRM on employee resilience is transmitted first through fairness, then through identification, pride and respect. Specifically, employees evaluate the treatment they experience in organizations applying C-HRM as fair, and then they infer their identity and status by perceived overall fairness. Perceived overall fairness gives employees confidence that they are working in a prestigious organization, signals to employees that they are valued and supported, and promotes the integration of the individual self-concept with the organization. Throughout the whole process of social identify, since individuals use the feedback obtained from the organization to create and maintain a positive social identity, they recognize that their value stems from their organizational membership. As a result, they are willing to exert themselves to improve the organizational status, which confers motivation on them to improve employee resilience. In short, C-HRM enhances perceived overall fairness, which invokes employees’ identification, pride, and respect in the organization, ultimately contributing to employee resilience. Therefore, we propose the following hypotheses:

Hypothesis 4a: C-HRM has an indirect relationship with employee resilience through perceived overall fairness via identification.

Hypothesis 4b: C-HRM has an indirect relationship with employee resilience through perceived overall fairness via pride.

Hypothesis 4c: C-HRM has an indirect relationship with employee resilience through perceived overall fairness via respect.

## 3 Method

To test the proposed hypotheses, two separate studies were conducted. Specifically, Study 1 collected data from a field survey in a large hospitality corporation in South China to test the impact of C-HRM on perceived overall fairness and the mediating role of perceived overall fairness in the relationship between C-HRM and the three dimensions of social identity (i.e., Hypotheses 1, 2a, 2b and 2c). Study 2 collected two-wave time-lagged data from an online survey in the hospitality industry to replicate the findings of Study 1 (i.e., Hypothesis 1, 2a, 2b and 2c) and further examine the impact of the three dimensions of social identity on employee resilience (i.e., Hypotheses 3a, 3b and 3c) and the serial mediating role of perceived overall fairness and the three dimensions of social identity in the relationship between C-HRM and employee resilience (i.e., Hypotheses 4a, 4b and 4c).

### 3.1 Study 1

#### 3.1.1 Sample and procedure

In Study 1, we tested Hypothesis 1 and Hypotheses 2a-2c by collecting data from eight branches under a large hospitality corporation in South China. We received support from the top executives of the company, who allowed us to launch a paper-and-pencil survey to their frontline employees. Our research team was present onsite to monitor the data collection process. With the assistance of the HR director, we randomly selected 395 frontline employees and distributed questionnaires to them. All questionnaires in envelopes were distributed and collected by the researchers during the workday. As a result, 342 questionnaires were returned by frontline employees and were included for further analyses. Among the frontline employee respondents, 64.3% of the respondents were females. The average age of the respondents was 27.8 years old. 36.8% of the respondents had a work tenure of below half a year, 23.1% of the respondents had a work tenure of between six months and one year, and 40.1% of the respondents had a work tenure of more than one year.

#### 3.1.2 Measurement

We chose the measurement variables from the existing literature. All measurements included in the questionnaire were the scales widely validated and used in previous studies. Since the data were collected in China, all questionnaires were presented and filled out in Chinese. The translation and back-translation method (Brislin, 1970) was adopted to ensure the accuracy of the translation of measurement items from English to Chinese. All variables (i.e., C-HRM, perceived overall fairness and social identity) were scored in the survey with a 5-point Likert-type scale ranging from 1 (strongly disagree) to 5 (strongly agree).

C-HRM: We measured C-HRM by a six-item scale from Li et al. (2012). A sample item was “The pay and bonus system in this organization is designed to maximize collectivism”. The Cronbach’s alpha was 0.73.

Perceived overall fairness: We measured perceived overall fairness by a three-item scale selected from Ambrose and Schminke (2009). This scale consists of three items assessing the personal fairness experience and three items assessing the perceptions of others more generally. Therefore, following the previous research (Rodell et al., 2017), we only selected the former subscale in consideration of our focus on employees’ perception of how they were treated. A sample item was “In general, I can count on this organization to be fair”. The Cronbach’s alpha was 0.77.

Social identity: We measured social identity by a sixteen-item scale from Blader and Tyler (2009). This scale consists of three subscales, including identification, pride and respect. Sample items of identification subscale was “Working at my company is important to the way that I think of myself as a person,” and the Cronbach’s alpha was 0.69. Sample items of respect subscale was “My company is one of the best companies in its field,” and the Cronbach’s alpha was 0.79. Sample items of respect subscale was “My managers respect the work I do,” and the Cronbach’s alpha was 0.82.

Control variables: We controlled employee’s gender and age. Gender was measured by a dummy variable as 0 (female) and 1 (male). Age was measured by the actual years old.

#### 3.1.3 Confirmatory factor analysis

Before testing the hypotheses, confirmatory factor analyses (CFAs) were performed to examine the convergent and discriminant validity of the variables. First of all, we tested a five-factor model that included C-HRM, perceived overall fairness, identification, pride, and respect. Fit indices including the root-mean-square error of approximation (RMSEA), comparative fit index (CFI) and Tucker-Lewis Index (TLI) were used to evaluate the overall fit of the model. As shown in Table 1, the five-factor model had an acceptable model fit (χ^2^ = 576.32, df = 265, *p* < 0.01, RMSEA = 0.06, CFI = 0.92, TLI = 0.91). Additionally, all factor loadings were significant, indicating the convergent validity. Then, we conducted CFAs on alternative four-factor models by randomly combining two variables and compared their model fit with that of the five-factor model to verify the model’s discriminant validity. The five-factor model fits the data better than any alternative four-factor models. Therefore, the discriminant validity was confirmed, and these five constructs were included in further analyses.

**TABLE 1:** Study 1: Results of confirmatory factor analysis for the measures of the study variables.

	χ^2^	df	CFI	TLI	RMSEA	Δdf	Δχ^2^
Five-factor model	576.32	265	0.92	0.91	0.06		
**Alternative four-factor models**							
Respect and identification combined	702.47	269	0.91	0.90	0.07	4	126.15**
Identification and pride combined	636.24	269	0.91	0.91	0.06	4	59.92**
Pride and respect combined	780.90	269	0.90	0.90	0.08	4	204.58**
C-HRM and perceived overall fairness combined	720.21	269	0.91	0.90	0.07	4	143.89**

**p < 0.01. CFI is the comparative fit index, TLI is the Tucker-Lewis Index and RMSEA is the root-mean-square error of approximation.

#### 3.1.4 Descriptive statistics

Table 2 presents the mean, standard deviation and zero-order Pearson correlation of the study variables in Study 1. The results demonstrated that C-HRM was positively correlated with perceived overall fairness (*r* = 0.45, *p* < 0.01), identification (*r* = 0.44, *p* < 0.01), pride (*r* = 0.49, *p* < 0.01), and respect (*r* = 0.45, *p* < 0.01). In addition, perceived overall fairness was positively correlated with identification (*r* = 0.29, *p* < 0.01), pride (*r* = 0.42, *p* < 0.01), and respect (*r* = 0.37, *p* < 0.01). These results are consistent with our hypotheses.

**TABLE 2:** Study 1: Means, standard deviations, and correlations.

	M	SD	1	2	3	4	5	6
1. Gender	0.36	0.48						
2. Age	27.80	7.92	0.18**					
3. C-HRM	4.02	0.61	0.01	0.04				
4. Perceived overall fairness	4.09	0.78	0.04	−0.11	0.45**			
5. Identification	3.63	0.74	0.05	0.15**	0.44**	0.29**		
6. Pride	3.92	0.74	−0.08	0.04	0.49**	0.42**	0.55**	
7. Respect	3.75	0.70	0.14*	0.19**	0.45**	0.37**	0.50**	0.51**

*N* = 342. *p < 0.05,

**p < 0.01.

#### 3.1.5 Hypotheses testing

Hypothesis 1 postulates that C-HRM has a positive impact on perceived overall fairness. To test this hypothesis, we regressed C-HRM on perceived overall fairness with the control variables. As shown in Table 3, the results revealed that after including the control variables in the regression analysis, C-HRM was significantly positively related to perceived overall fairness (β = 0.58, *p* < 0.001, model 2). Therefore, Hypothesis 1 was supported.

**TABLE 3:** Study 1: Results of regression analysis.

	Perceived overall fairness	
**Variables**	**Model 1**	**Model 2**
Intercept	4.37*** (0.15)	2.08*** (0.28)
**Control variables**		
Gender	0.10 (0.09)	0.10 (0.08)
Age	−0.01* (0.01)	−0.01** (0.01)
**Independent variable**		
C-HRM		0.58*** (0.06)
*R* ^2^	0.02	0.22
Δ*R*^2^	0.02	0.21***

*N* = 342. The numbers in brackets are robust standard errors.

*p < 0.05,

**p < 0.01,

***p < 0.001.

Hypotheses 2a, 2b and 2c predict the indirect effects between C-HRM and the three dimensions of social identity (i.e., identification, pride and respect, respectively) via perceived overall fairness. To test these indirect effects, we conducted bootstrapping analyses using 5,000 re-samples by Model 4 of PROCESS 3.5 (Hayes, 2017). The PROCESS macro is able to test a specific indirect effect when controlling for the effects of all other mediators (Preacher and Hayes, 2008). The indirect effect of the simple mediating effect is supported if the bias-corrected confidence interval does not contain zero at 95% level of confidence, after controlling for the effects of the other mediators (Hayes, 2017). As shown in Table 4, results of bootstrapping analysis indicated that the indirect effect between C-HRM and identification via perceived overall fairness was significant (effect = 0.07, SE = 0.04, bias-corrected confidence interval = 0.001, 0.145), supporting Hypothesis 2a. The indirect effect between C-HRM and pride via perceived overall fairness also was significant (effect = 0.14, SE = 0.04, bias-corrected confidence interval = 0.069, 0.224), supporting Hypothesis 2b. Moreover, the indirect effect between C-HRM and respect via perceived overall fairness was significant (effect = 0.12, SE = 0.04, bias-corrected confidence interval = 0.049, 0.194), supporting Hypothesis 2c.

**TABLE 4:** Study 1: Bootstrapping analysis for the indirect effects of simple mediating effects.

Mediator: perceived overall fairness	Effect	SE	LLCI	ULCI
**Dependent variable**				
Identification	0.07	0.04	0.001	0.145
Pride	0.14	0.04	0.069	0.224
Respect	0.12	0.04	0.049	0.194

The bias-corrected confidence intervals were based on 5,000 re-samples at the 95% level of confidence.

### 3.2 Study 2

To replicate the findings of Study 1 (i.e., Hypothesis 1 and Hypotheses 2a-2c), and to further test Hypotheses 3a-3c and Hypotheses 4a-4c, we conducted Study 2 for an online survey in the hospitality industry.

#### 3.2.1 Sample and procedure

To further ensure that the sample is representative, sufficient and random, we recruited front-line employees who volunteered to participate in Study 2 in the hospitality industry from all over China by a Chinese online survey platform (WJX). It is getting increasingly popular to recruit participants by an online survey platform in hospitality research (e.g., Haldorai et al., 2023; Zientara et al., 2023). To avoid common method variance, we collected two-wave data. Specifically, C-HRM and perceived overall fairness and were collected in the first wave, and a total of 490 respondents returned the questionnaires. Two weeks later, respondents participating in the first wave were given a second questionnaire to measure their social identify and employee resilience. Finally, 294 paired questionnaires were included in the analysis, with a response rate of 60.0%. In the sample, 61.9% were female, 14.6% were under the age of 25, 70.4% were aged 26–35, 12.2% were aged 36–45 and only 2.7% were over the age of 46. 40.1% of the respondents had a bachelor degree.

#### 3.2.2 Measurement

All variables and measurements included in the questionnaire were chosen from the existing literature. The survey was proceeded in China and all questionnaires were presented in Chinese. Thus, we abided by a translation and back-translation method to ensure the accuracy of the scale items in Chinese (Brislin, 1970). All variables (i.e., C-HRM, perceived overall fairness, social identity, and employee resilience) were measured by a 5-point Likert-type scale ranging from 1 = strongly disagree to 5 = strongly agree.

C-HRM and perceived overall fairness were measured using the same scales in Study 1. The Cronbach’s alpha were 0.61 and 0.68, respectively.

Social identity: Social identity was measured by the same scale used in Study 1 (Blader and Tyler, 2009). The Cronbach’s alpha of identification, pride and respect were 0.69, 0.81 and 0.75, respectively.

Employee resilience: Employee resilience was measured using a six-item scale from Smith et al. (2008). A sample item was “It does not take me long to recover from a stressful event”. The Cronbach’s alpha was 0.81.

Control variables: We controlled for frontline employee’s gender, age and education level. Gender was measured as 1 indicating male and 2 indicating female. Age was measured as 1 (under 25 years old), 2 (26–35 years old), 3 (36–45 years old high school), 4 (46–55 years old) and 5 (56 years old and above). Education level was measured as 1 (junior high school and below), 2 (high school or vocational school), 3 (associate degree), 4 (university degree) and 5 (postgraduate and above).

#### 3.2.3 Confirmatory factor analysis

Table 5 presents the results of the confirmatory factor analyses (CFAs) in Study 2. The six-factor model (i.e., including all the variables of C-HRM, perceived overall fairness, identification, pride, respect and employee resilience) had an acceptable model fit (χ^2^ = 626.49, df = 419, *p* < 0.01, RMSEA = 0.04, CFA = 0.93, TLI = 0.92). Moreover, all the factor loadings were significant, indicating the convergent validity. Additionally, we performed CFAs on the several alternative five-factor models derived from a random combination of two variables collected during the same time period (i.e., either collected in the first wave or the second wave). The results indicated that the six-factor model showed superior fit compared to any of the alternative models, confirming the discriminant validity. Therefore, all the variables were retained for further analysis.

**TABLE 5:** Study 2: Results of confirmatory factor analysis for the measures of the study variables.

	χ^2^	df	CFI	TLI	RMSEA	Δdf	Δχ^2^
Six-factor model	626.49	419	0.93	0.92	0.04		
**Alternative five-factor models**							
Respect and identification combined	661.79	424	0.92	0.91	0.04	5	35.3**
Identification and pride combined	637.55	424	0.93	0.92	0.04	5	11.06*
Pride and respect combined	645.97	424	0.92	0.92	0.04	5	19.48**
C-HRM and perceived overall fairness combined	683.16	424	0.91	0.90	0.05	5	56.67**
Identification and employee resilience combined	722.94	424	0.91	0.90	0.05	5	96.45**
Pride and employee resilience combined	753.84	424	0.91	0.90	0.05	5	127.35**
Respect and employee resilience combined	745.03	424	0.91	0.90	0.05	5	118.54**

*p < 0.05,

**p < 0.01.

CFI is the comparative fit index, TLI is the Tucker-Lewis Index and RMSEA is the root-mean-square error of approximation.

#### 3.2.4 Descriptive statistics

Table 6 presents the descriptive statistics for all study variables. As shown, C-HRM was positively correlated with perceived overall fairness (*r* = 0.45, *p* < 0.01), identification (*r* = 0.53, *p* < 0.01), pride (*r* = 0.57, *p* < 0.01), respect (*r* = 0.49, *p* < 0.01) and employee resilience (*r* = 0.40, *p* < 0.01). Perceived overall fairness was positively correlated with identification (*r* = 0.43, *p* < 0.01), pride (*r* = 0.55, *p* < 0.01), respect (*r* = 0.62, *p* < 0.01) and employee resilience (*r* = 0.42, *p* < 0.01). Identification (*r* = 0.59, *p* < 0.01), pride (*r* = 0.61, *p* < 0.01) and respect (*r* = 0.62, *p* < 0.01) were positively correlated with employee resilience. These results are in line with our hypotheses.

**TABLE 6:** Study 2: Means, standard deviations, and correlations.

	Mean	SD	1	2	3	4	5	6	7	8
1. Gender	1.62	0.49								
2. Age	2.03	0.62	−0.04							
3. Education	3.40	0.57	−0.04	0.13*						
4. C-HRM	3.94	0.46	−0.07	0.11	0.07					
5. Perceived overall fairness	4.10	0.59	−0.10	0.11	0.11	0.45**				
6. Identification	3.89	0.59	−0.22**	0.07	0.07	0.53**	0.43**			
7. Pride	3.86	0.67	−0.16**	0.14*	0.05	0.57**	0.55**	0.76**		
8. Respect	3.95	0.55	−0.13*	0.14*	0.09	0.49**	0.62**	0.69**	0.78**	
9. Employee resilience	3.72	0.66	−0.12*	0.09	0.10	0.40**	0.42**	0.59**	0.61**	0.62**

*N* = 294.

*p < 0.05,

**p < 0.01.

#### 3.2.5 Hypotheses testing

To test Hypothesis 1, which predicts that C-HRM positively affect perceived overall fairness, regression analysis of C-HRM on perceived overall fairness was performed with the control variables. As the results in Table 7 shown, after controlling for the control variables in the regression analysis, C-HRM was significantly positively related to perceived overall fairness (β = 0.56, *p* < 0.001, model 2). Therefore, Hypothesis 1 was supported.

**TABLE 7:** Study 2: Results of regression analysis of C-HRM on perceived overall fairness.

	Perceived overall fairness	
Variables	Model 1	Model 2
Intercept	3.76*** (0.25)	1.68*** (0.34)
**Control variables**		
Gender	−0.11 (0.07)	−0.07 (0.06)
Age	0.09 (0.06)	−0.04 (0.05)
Education	0.10 (0.06)	0.07 (0.05)
**Independent variable**		
C-HRM		0.56*** (0.07)
*R* ^2^	0.03	0.22
Δ*R*^2^	0.03*	0.19***

*N* = 294. The numbers in brackets are robust standard errors.

*p < 0.05,

***p < 0.001.

To test Hypotheses 2a, 2b and 2c, which postulate that perceived overall fairness mediates the relationships between C-HRM and the three dimensions of social identity (i.e., identification, pride and respect, respectively), bootstrapping analyses using 5,000 re-samples were performed by Model 4 of PROCESS 3.5 (Hayes, 2017). Table 8 summarizes results of bootstrapping analysis for the indirect effects of simple mediating effects. As shown, results of bootstrapping analysis demonstrated that the indirect effect between C-HRM and identification through perceived overall fairness was significant (effect = 0.13, SE = 0.04, bias-corrected confidence interval = 0.056, 0.212). Moreover, the indirect effects between C-HRM and pride (effect = 0.23, SE = 0.05, bias-corrected confidence interval = 0.141, 0.329), respect (effect = 0.26, SE = 0.05, bias-corrected confidence interval = 0.177, 0.351) via perceived overall fairness were also significant. Therefore, Hypotheses 2a, 2b and 2c were supported.

**TABLE 8:** Study 2: Bootstrapping analysis for the indirect effects of simple mediating effects.

Mediator: perceived overall fairness	Effect	SE	LLCI	ULCI
**Dependent variable**				
Identification	0.13	0.04	0.056	0.212
Pride	0.23	0.05	0.141	0.329
Respect	0.26	0.05	0.177	0.351

The bias-corrected confidence intervals were based on 5,000 re-samples at the 95% level of confidence.

Hypotheses 3a, 3b and 3c propose that identification, pride and respect have a positive effect on employee resilience, respectively. To test those hypotheses, we conducted a regression analysis of identification, pride and respect on employee resilience with the control variables. As presented in Table 9, results showed that after including the control variables in the linear-regression, identification (β = 0.25, *p* < 0.01, model 2), pride (β = 0.19, *p* < 0.05, model 2) and respect (β = 0.38, *p* < 0.001, model 2) were all significantly and positively related to employee resilience, supporting Hypotheses 3a, 3b and 3c.

**TABLE 9:** Study 2: Results of regression analysis of identification, pride, and respect on employee resilience.

	Employee resilience	
Variables	Model 1	Model 2
Intercept	3.49*** (0.29)	0.35 (0.31)
**Control variables**		
Gender	−0.15 (0.08)	0.00 (0.06)
Age	0.07 (0.06)	−0.01 (0.05)
Education	0.10 (0.07)	0.05 0 (0.05)
**Independent variable**		
Identification		0.25** (0.08)
Pride		0.19* (0.08)
Respect		0.38*** (0.09)
*R* ^2^	0.03	0.45
Δ*R*^2^	0.03*	0.42***

*N* = 294. The numbers in brackets are robust standard errors.

*p < 0.05,

**p < 0.01,

***p < 0.001.

Hypotheses 4a, 4b and 4c propose that perceived overall fairness and the three dimensions of social identity (i.e., identification, pride and respect, respectively) play a serial mediating role in the indirect effect of C-HRM on employee resilience. To test those hypotheses, we performed bootstrapping analysis using 5,000 re-samples by Model 81 of PROCESS 3.5 (Hayes, 2017). As the Table 10 shown, the indirect effect between C-HRM and employee resilience through perceived overall fairness and identification (effect = 0.03, SE = 0.02, bias-corrected confidence interval = 0.007, 0.072), through perceived overall fairness and respect (effect = 0.09, SE = 0.03, bias-corrected confidence interval = 0.035, 0.163) were significant, supporting Hypotheses 4a and 4c. In addition, the indirect effect between C-HRM and employee resilience via perceived overall fairness and pride (effect = 0.04, SE = 0.02, bias-corrected confidence interval = −0.005, 0.090) was not significant. Therefore, Hypothesis 4b was not supported.

**TABLE 10:** Study 2: Bootstrapping analysis for the indirect effects of chain mediating effects.

Dependent variable: employee resilience	Effect	SE	LLCI	ULCI
**Chain mediating effects through**				
Perceived overall fairness and identification	0.03	0.02	0.007	0.072
Perceived overall fairness and pride	0.04	0.02	−0.005	0.090
Perceived overall fairness and respect	0.09	0.03	0.035	0.163

The bias-corrected confidence intervals were based on 5,000 re-samples at the 95% level of confidence.

## 4 Results

Table 11 presents results of hypotheses testing through regression analyses and bootstrapping analyses in Study 1 and Study 2. Specifically, the results of Study 1 supported Hypotheses 1, 2a, 2b and 2c. The results of Study 2 replicated the results of Study 1 (i.e., Hypotheses 1, 2a, 2b and 2c). In addition, the results of Study 2 further supported Hypotheses 3a, 3b, 3c, 4a and 4c, and Hypothesis 4b was not supported.

**TABLE 11:** Summary of hypotheses testing results.

Hypotheses	Testing results	
	Study 1	Study 2
Hypothesis 1: C-HRM is positively related to perceived overall fairness.	Supported	Supported
Hypothesis 2a: Perceived overall fairness mediates the relationship between C-HRM and identification.	Supported	Supported
Hypothesis 2b: Perceived overall fairness mediates the relationship between C-HRM and pride.	Supported	Supported
Hypothesis 2c: Perceived overall fairness mediates the relationship between C-HRM and respect.	Supported	Supported
Hypothesis 3a: Identification is positively related to employee resilience.	–	Supported
Hypothesis 3b: Pride is positively related to employee resilience.	–	Supported
Hypothesis 3c: Respect is positively related to employee resilience.	–	Supported
Hypothesis 4a: C-HRM has an indirect relationship with employee resilience through perceived overall fairness via identification.	–	Supported
Hypothesis 4b: C-HRM has an indirect relationship with employee resilience through perceived overall fairness via pride.	–	Not supported
Hypothesis 4c: C-HRM has an indirect relationship with employee resilience through perceived overall fairness via respect.	–	Supported

## 5 Discussion

By drawing on the perspective of group engagement model (Tyler and Blader, 2003) and integrating work on C-HRM (Li et al., 2012) and employee resilience (McEwen and Boyd, 2018), this study originally examines the relationship between C-HRM implemented by an organization and employee resilience of frontline employees in the hospitality industry and the chain mediating effect of perceived overall fairness and social identity. The findings of two separate studies provide empirical evidence for the proposed hypotheses in our theoretical model.

As the results of Study 1 and Study 2 both revealed, C-HRM has a significant positive effect on perceived overall fairness of frontline employees in the hospitality industry (Hypothesis 1). C-HRM is a highly oriental management issue, especially in organizations in the Chinese context, where collectivist cultural values are deeply rooted due to the influence of traditional Chinese culture. C-HRM advocates that employees ensure harmony with others by aligning themselves with the rest of the organization, emphasizing a focus on interpersonal similarity and team consistency over individual specificity. Therefore, employees influenced by C-HRM may ignore differences and inequalities between individuals, thus contributing to perceived overall fairness.

The results of Study 1 and Study 2 also show that perceived overall fairness mediates the relationship between C-HRM and the three dimensions of social identity (Hypotheses 2a, 2b, and 2c). Drawing on the group engagement model (Tyler and Blader, 2003), fairness conveys important information about identity and status. Employees who are treated fairly in the organization perceive their organization has a high social status as well as they have a high status in the organization, and integrate their self with the organization to build a positive self-concept. Therefore, C-HRM has a positive indirect effect on identification, pride and respect through perceived overall fairness.

Furthermore, the results of Study 2 confirm that identification, pride and respect have a significant positive effect on employee resilience (Hypotheses 3a, 3b, and 3c). Since organizational membership can enhance or diminish an individual’s self-image and value, motivated by the desire to maintain and enhance their self-concept, employees will actively participate in group engagement and invest more effort in promoting organizational success. Therefore, the degree of identification with the organization (i.e., identification), the evaluation of organizational prestige (i.e., pride), and the judgment of self-worth (i.e., respect) all endow employees with greater resilience, which enables employees to collect, integrate, and utilize organizational resources when facing work challenges, seek opportunities for continuous improvement to achieve personal and organizational goals, and adapt quickly to adversity.

The results of Study 2 also reveal that C-HRM has a positive indirect effect on employee resilience through perceived overall fairness via identification (Hypothesis 4a). C-HRM also has a positive indirect effect on employee resilience through perceived overall fairness via respect (Hypothesis 4c). However, the serial mediating effect of perceived overall fairness and pride between C-HRM and employee resilience was not statistically significant (Hypothesis 4b). We suggest that a possible reason is that the samples for this study are drawn from frontline employees working in the hospitality industry. For such groups, whose external social status and prestige is generally low due to the fact that their job content includes mostly low-skill and mechanized labor, more importance may be attached to the internal status within the organization. Therefore, respect that frontline employees in the hospitality industry experience in their organizations may be more closely related to emotions, attitudes, and behaviors than pride. Furthermore, previous research indicates that although both pride and respect represent organization-related status, respect may be more significant than pride (Seta and Seta, 1996; Fuller et al., 2009).

## 6 Conclusion

Drawing on the group engagement model, this study proposes a serial mediation model to understand the significance of C-HRM, perceived overall fairness, and social identity in predicting employee resilience. Part of the hypothesized relationships were supported by the empirical data collected from a field survey and a two-wave online survey in the hospitality industry in China. Based on the results, we draws the following conclusions: (1) C-HRM has a positive impact on perceived overall fairness; (2) perceived overall fairness plays a mediating role between C-HRM and the three dimensions of social identity (i.e., identification, pride and respect); (3) the three dimensions of social identity (i.e., identification, pride and respect) can foster employee resilience; (4) perceived overall fairness and identification, respect play a serial mediating role in the relationship between C-HRM and employee resilience. These results provide Asian HRM and international HRM with valuable theoretical significance and practical implications.

### 6.1 Theoretical contributions

This study contributes to theory in several ways. First, this study enriches literature on C-HRM by investigating new positive outcomes of C-HRM. Although C-HRM has attracted the attention of strategic HRM scholars, relevant research is still limited and mainly focus on how C-HRM may improve employee creativity and organizational performance (e.g., Li et al., 2012; Chen et al., 2021). Little is known about the positive effects of C-HRM on the positive psychology and wellbeing of employees. This study examines the beneficial role of C-HRM in promoting employee resilience at work and its underlying mechanism, which has not been explored in previous studies. Therefore, this study not only enriches research on C-HRM, but also contributes to oriental management literature and international HRM theories.

Second, this study contributes to the research on employee resilience by identifying new antecedents of employee resilience. Previous studies mainly focused on the definition of “resilience,” and most of them were descriptive and qualitative studies, while few empirical studies focused on the antecedents of the resilience of hospitality frontline employees. This study identifies C-HRM as one of the antecedents of employee resilience and provides empirical evidence from frontline employees in the hospitality industry. Therefore, this study enriches the research on the antecedents of employee resilience.

Third, this study enriches the theoretical perspective of understanding the development of employee resilience by exploring the influencing factors of employee resilience from the perspective of the group engagement model. Most previous studies explored the factors that promote employee resilience from the perspective of social exchange (e.g., Mao et al., 2023) or resource conservation (e.g., Cai et al., 2023). Different from studies that focus on social exchange and work resources, this study is based on the group engagement model and puts perceived overall fairness and social identity at the core to explain the role of C-HRM on employee resilience. Therefore, this study provides a new theoretical perspective for understanding the improvement of employee resilience, and provides forceful support for predicting employees’ key job capabilities by the group engagement model.

Lastly, this study enriches the research on social identity by emphasizing the multidimensional structure of social identity, and paying attention to both the cognitive and evaluative components of social identity. Most of prior studies on social identity focus on its cognitive component (i.e., identification), few studies embody the three dimensions of social identity. In other words, the evaluative components (i.e., pride and respect) are often overlooked in organizational research. In the process of exploring the explicatory mechanism of the impact C-HRM has on employee resilience, this study incorporates pride and respect into the process of social identity. Therefore, this study is helpful to understand social identity in a more accurate and comprehensive approach.

### 6.2 Practical implications

The study also offers insights of relevance to business managers, especially hospitality practitioners. First, this study points out useful measures for organizations to foster employee resilience among employees by explores the antecedents to enhance the employee resilience. Given the importance of employee resilience in job satisfaction and performance, organization managers hope to promote employee resilience through effective management practices, thereby improving the core competitiveness of the organization. This study empirically confirms that C-HRM can be fundamental for the cultivation of employee resilience. Furthermore, the serial mediating role of perceived overall fairness and the two dimensions of social identity (i.e., identification and respect) suggests that organizations should continuously improve HRM practices fertilizing organizational justice that could increase individual sense of identification and respect as a part of the organization, so as to help s/he maintain a good psychological state and self-improvement motivation to actively cope with difficulties and changes. Specifically, organizations should pay attention to creating an equitable work environment through transparent management communications, fair pay practices, and respectful interpersonal interactions among members.

Second, this study provides guidance for organizations to establish effective human resource management system by alerting the vital influence of C-HRM within the Chinese cultural context. Especially for organizations operating in collectivistic cultures, such as China, Japan, and South Korea, understanding the integration of collectivism with strategic HRM is critical. The findings highlight the significant positive effects of C-HRM on perceived overall fairness, social identity, and employee resilience. Therefore, organizational managers should consider implementing C-HRM to improve organizational fairness, and encourage employees to establish a strong connection with the organization, thereby guiding them to make greater contributions to the organization. These include setting appropriate and clear organizational goals, integrating team development programs into employee training, hiring employees with a high degree of team spirit and organizational loyalty, and establishing clear criteria to reward team contributions.

Finally, this study emphasizes the benefits of cultivating employee social identity, and provides practical suggestions on how to improve employee social identity. Social identity is a key driver of employee behavior, as employees with a strong social identity in an organization are generally more loyal, engaged, and have a stronger motivation to improve themselves, thus becoming resilient employees. We recommend that organizational managers take appropriate management interventions to ensure that employees have a high level of social identity within the organization. Specifically, organizations should adopt practices emphasizing teamwork and organizational development to strengthen cohesion and foster a sense of identification with the organization. Organizations can also build a positive social image by showing integrity and social responsibility, leading employees to take pride in the organization. Besides, organizations show concern for the personal value of employees, establish a support network, and supply career development planning and internal promotion opportunities to enhance their sense of being respected in the organization.

## 7 Limitations and future research directions

Despite the meaningful contributions of this study to academic research and management practice, there are still some limitations that offer promising directions for future research. First, since we applied a cross-sectional design with data collected from a field survey and an online questionnaire to test the hypothetical model, causality between study variables cannot be verified. Therefore, future research should adopt longitudinal study design or experiment to verify the causality. Additionally, Cronbach’s α of a few of the measures in this research was greater than 0.6 and less than 0.7. Although this is acceptable (e.g., Lam et al., 2023; Waltré et al., 2023; Gauglitz and Schyns, 2024), the reliability of the measurement tools should be tested again in future research.

Second, this study only considers perceived overall fairness as the mediating variable between C-HRM and social identity. This study has confirmed that C-HRM emphasizes team consistency and similarity, reducing the perception of differences among individuals and improving the perception of fairness. Employees further utilize fairness as the basis for evaluating personal social identity. However, the results of bootstrap analysis in Study 1 and Study 2 both show that perceived overall fairness partially mediates the relationship between C-HRM and social identity. In other words, there are other mediating factors between the two variables that have not been addressed in this study and need to be further explored. In addition, it is plausible that an overemphasis on collectivism in HRM may invoke counterproductive effects, such as “free rider” or “communal pot,” which can undermine employee motivation and initiative. Therefore, future research should further explore other outcomes of C-HRM and its potential mechanisms.

Third, the external validity of this study has yet to be tested. This study is only conducted in China, a country with a highly collectivistic orientation. It remains uncertain whether the findings can be generalized to organizations in other cultural contexts. Therefore, future research should further verify whether the positive effects of C-HRM can be replicated to other cultures (e.g., individualistic culture) to test the cross-cultural applicability of C-HRM.

## Data availability statement

The original contributions presented in this study are included in the article/supplementary material, further inquiries can be directed to the corresponding author.

## Ethics statement

The studies involving humans were approved by the Ethics Committee of Shanghai University. The studies were conducted in accordance with the local legislation and institutional requirements. The participants provided their written informed consent to participate in this study.

## Author contributions

SC: Conceptualization, Data curation, Investigation, Methodology, Visualization, Writing – original draft, Writing – review & editing. XH: Data curation, Funding acquisition, Investigation, Methodology, Project administration, Resources, Software, Supervision, Writing – review & editing. YX: Investigation, Writing – review & editing. YW: Investigation, Writing – review & editing.
